# A scoping review on current technology-based approaches to support breastfeeding and informal human milk exchange practices

**DOI:** 10.1371/journal.pone.0290311

**Published:** 2023-09-14

**Authors:** Siti Fatimah Abdul Razak, Nur Liyana Rosli, Noor Hisham Kamis, Norsyamlina Che Abdul Rahim, Mohd Fikri Azli Abdullah

**Affiliations:** 1 Faculty of Information Science and Technology, Multimedia University, Ayer Keroh, Melaka, Malaysia; 2 Faculty of Hospitality, Tourism and Wellness, Universiti Malaysia Kelantan, Kelantan, Malaysia; Sefako Makgatho Health Sciences University, SOUTH AFRICA

## Abstract

Informal human milk exchange is the practice of donating and receiving expressed human milk based on mutual consent between the donor and receiver in the need of human milk for infants below 2 years old. Main concerns related to informal human milk exchange is related to milk siblings and safety handling of the expressed breastmilk. Even though there are countries which have policies and procedures related to human milk bank, informal milk exchange has not been given much attention. Compared to human milk bank, informal human milk exchange is not regulated. This study aims to identify the system focused on personalized breastfeeding tracking and monitoring, online discussion forum, web-based consultation, and breastfeeding station locator. Review of current applications in supporting breastfeeding practices was conducted based on the PRISMA-ScR framework. A literature search was conducted in Scopus and Google Scholar databases to identify articles published in English or Malay and containing systems/applications related to breastfeeding, milk sharing, milk exchange, milk siblings/kinship within the societal context. According to the scoping review, current scientific publications mostly focused on breast milk, breastfeeding, and milk banking concerns, with recurring themes including social reasons, lactation insufficiency, and unsolved nursing problems. These themes highlight the complexities and complexities of informal human milk exchange practices. Two reviewers screened the articles, and the data were extracted and narratively synthesized. During the primary database search, 360 articles were found based on the related titles, abstracts, and keywords. Seventy eight met the inclusion criteria and were finalized in this review. We found that most scholarly works focused on breast milk, breastfeeding and milk banking challenges and issues with recurrent themes i.e., societies, lactation inadequacy and unresolved nursing problems. Based on our literature search and to the best of our knowledge, there is no recent scoping reviews which focuses on technology-based approaches on informal human milk exchange. Findings from this scoping review is important for advancing research and practice in this field, as well as improving outcomes for individuals and families affected by informal human milk exchange.

## Introduction

It is a well-known fact that human milk is full of essential vitamins and nutrients, as well as antibodies that provide immunization and protect infants’ health. According to the World Health Organisation (WHO), breastmilk should be the only consumption during infants’ first six months. However, not all infants are privileged to be breastfed by their biological mothers. Mothers who have low breast milk supply or undergo a medical treatment which is not safe to breastfeed may be inclined to milk exchange or sharing practice [1]. In a situation where the biological mother is deceased after giving birth to her child, relatives of a deceased mother may also choose to provide breastmilk instead of milk formula to the needy infant. In milk exchange practice, infants are connected to non-maternal mothers who are blessed to produce breast milk. Technology-based initiatives have emerged as promising solutions to promote and encourage breastfeeding and informal human milk exchange practises as cultural views alter. The existing landscape of technology-based approaches aim to improve breastfeeding practises, fostering milk sharing networks, and offering alternatives for needy infants.

Globally, there is an increase in the use of expressed human milk for infant feedings, whether through peer-to-peer human milk sharing or commercial markets for human milk and related products [2]. Informal milk exchange is normally practiced providing nutritious breastmilk to the infants where formal and informal channels have been used to facilitate the practice [3]. Since 2010, non-maternal nursing and breastmilk exchange is facilitated via websites and social media platforms [4,5]. Initial connection between the donor and recipient is through online group or self-agreement [6] among family members or close friends or wet nurse without monetary compensation, mostly to save an infant’s life or when the mother’s breastmilk supply is not sufficient. Participants need to be alert of the privacy settings and anonymity choices which vary among different ICT platforms. Furthermore, total anonymity is not always ensured, particularly on social media platforms where it generally has open structure and user profiles are frequently examined by others. Nevertheless, within two years, the widespread use of mobile social apps, internet-capable smartphones, and widespread internet usage in Western countries have resulted in more intense research in milk sharing.

In the context of milk kinship, studies have revealed that informal human milk exchange sharing is more socially and culturally acceptable in most communities where moral and ethical dilemma is a concern [2]. In a recent scoping review on key ideas supporting milk sharing research, it was concluded that milk sharing raises several important issues related to individual experience, emotional aspects of milk sharing and religious concerns [4]. Nevertheless, there are mothers who are reluctant or against the practice of giving and receiving breastmilk. Only 15.2% of mothers are positive towards milk exchange practice for fear of safety of the breastmilk and health-related fallacies [7]. Hence, it is critical to acknowledge that each mother’s decision is impacted by a variety of personal considerations and beliefs. Addressing some mothers’ worries is critical to creating a supportive and educated atmosphere in which evidence-based information may assist reduce anxieties and misconceptions. Researchers and healthcare professionals can facilitate open dialogues and promote comprehensive support systems that cater to the unique needs and preferences of breastfeeding mothers and those considering informal milk exchange practices by understanding and engaging with these varying viewpoints.

For Muslims, concern of milk mating is the main reason since it is prohibited in Islam. Given the religious ramifications and the importance of the Mahram idea in Islam, it is of utmost importance to Muslims that infants who are breastfed by the same women are prohibited from marriage with one another [8]. They are known as milk siblings when two requisites are fulfilled i.e., (i) the age of the child is not more than two years old, and (ii) the child has been breastfed at least five times. The testimony of two men or one man and two women can be used to prove the relationship among milk siblings. Hence, mothers of the children who share the same milk must be acquainted to prevent future instances of "accidental marriage" which will result in annulled marriage. This is clearly stated in the Quran (*Surah AnNisa’* 4:23) as follows:

“*Prohibited to you (for marriage) are your mothers*, *your daughters*, *your sisters*, *your father’s sisters*, *your mother’s sisters*, *your brother’s daughters and sister’s daughters*, *your (milk) mothers who nursed you*, *your sisters through nursing*, *your wives’ mothers*, *and your step-daughters under your guardianship (born) of your wives unto whom you have gone in*. *But if you have not gone in unto them*, *there is no sin upon you*. *And (also prohibited are) the wives of your sons who are from your (own) loins*, *and that you take (in marriage) two sisters simultaneously*, *except for what has already occurred”*.

Therefore, any technology-based approach which aims to support informal human milk exchange should provide choices for anonymity and privacy, religious preference filters, and instructional resources on the relevance of milk siblings. By taking these considerations into account, the ICT platform will be more appealing to nursing mothers and receivers who are looking for safe and culturally appropriate milk exchange agreements within the context of Islamic beliefs.

Moreover, knowing the donor or donors makes it easier to document where the human milk comes from and the relationships it fosters [2]. Prior to the human milk exchange, the families of the donor and beneficiary shall meet to discuss about compatibility, religious implications of kinship, and the ban on marriage between the recipient newborn and the donor’s offspring [9]. Even so, informal milk exchange has not been regulated to properly record the milk-child relationship [10]. Interaction between donors and recipients is frequently made easier not only by social networking sites centered online, but it is also made easier offline by direct connections among family, friends, and local community members. The permission of the Islamic authorities in Turkey, Saudi Arabia and Bangladesh with electronically saved data for future verification is still pending [9].

Nonetheless, breastfeeding mothers have applied different ways to remind themselves of their involvement in the informal milk exchange. This includes recording the breastfed child name and birth date in their diaries, keeping a copy of the breastfed child’s birth certificate and the identity card of the breastfed child’s biological parents, verbally informing their close relatives, keeping a photo of the breastfed child with the biological parents and siblings. However, there are also breastfeeding mothers who do not have any record and rely totally on their memory of the occasion and close relatives [11]. Hence, it is likely that families connected through informal milk sharing exchange will lose contact of the whereabouts of their breastfed child over time. The relationship becomes more complex when the size of the families grows. This is a highly concerning situation in which the milk siblings are not aware of the relationship formed during their infanthood. Hence, the impact would be high when the siblings get married because in Islamic law, they are prohibited to marry each other. For this reason, The European Council for Fatwa and Research (ECFR) imposed a 30-year record retainment policy for human milk bank to ensure the traceability of milk siblings and enabling the Muslims to determine whether milk kinship was present between two prospective spouses [9].

Based on our literature, we found that the most recent investigations of human milk sharing in Western, Educated, Industrialized, Religious and Democratic (WEIRD) societies, lactation inadequacy and unresolved nursing problems are recurrent themes. As such, amongst the initiatives proposed is to incorporate technologies to support breastfeeding practices. These technologies are offered in various mode and features which are generally divided into personalized breastfeeding tracking and monitoring, online discussion forum, web-based consultation, and breastfeeding station locator [12]. The approaches implemented aimed to change the breastfeeding behavior for effective breastfeeding practice. There is a very limited source of technologies or applications addressing the issue related to milk siblings. Not much attention has been given to this matter since most research on breastfeeding is from Western countries and focuses on the development of the human milk bank.

We hope that by conducting this scoping review, we can add to the growing body of knowledge about technology’s potential to revolutionise breastfeeding support and informal human milk exchange, promoting optimal health and well-being for infants and nurturing a supportive environment for breastfeeding mothers and recipients alike. This paper is organized into four sections. This Introduction section is followed by Section 2 which presents the methodology based on the PRISMA-ScR framework. Next, Section 3 presents the results and also discusses the findings. Lastly, Section 4 concludes this study.

## Methods

### Stage 1: Search strategy

**Fig 1:**
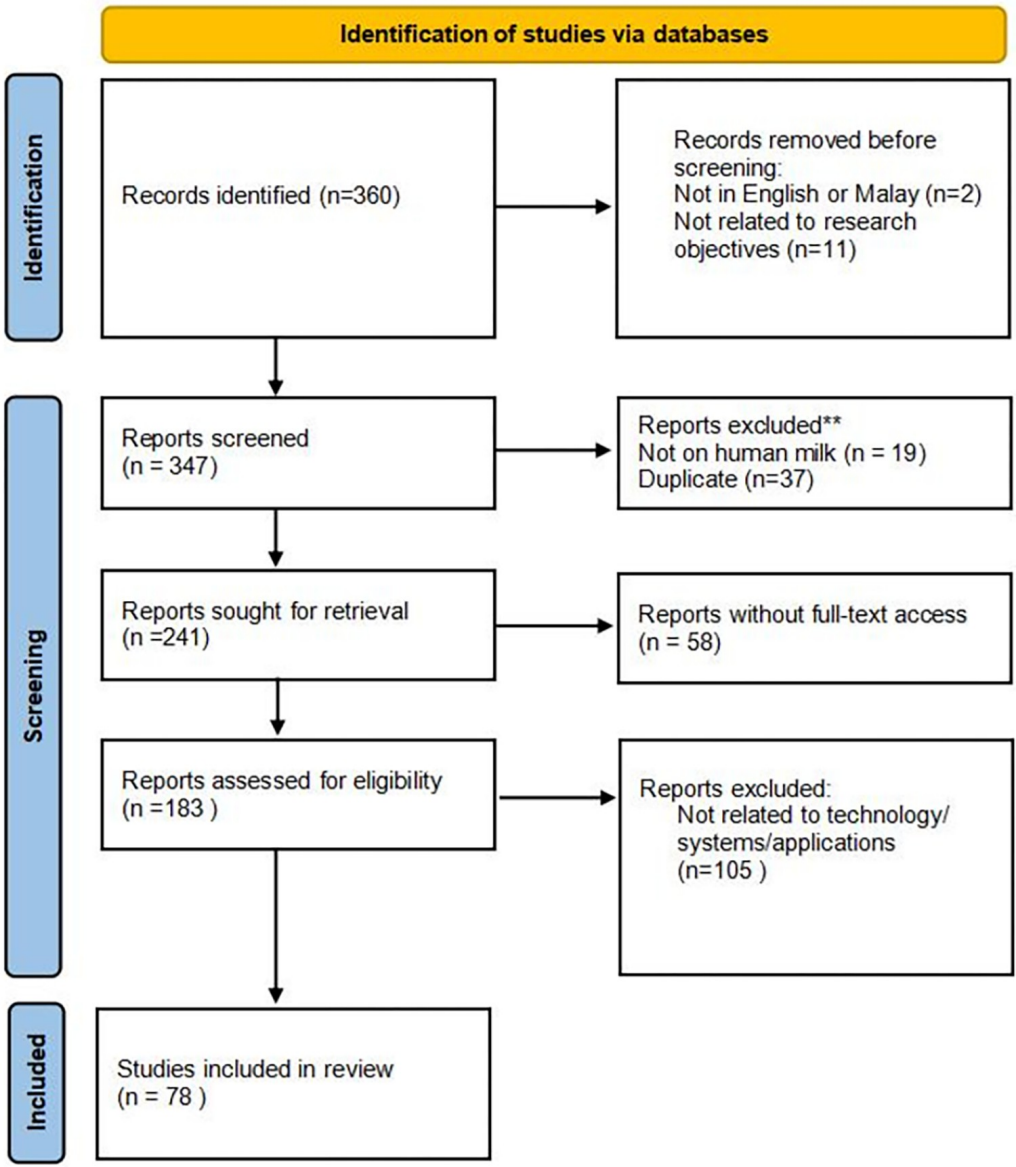
PRISMA-ScR framework.

The well-known electronic databases for scholarly work, i.e., Scopus and Google Scholar were utilized to retrieve relevant works to our study. Only documents published in English or Malay between 2018 and 2022 that matched the search strings i.e., “milk exchange”, “milk sharing”, “milk kinship”, “milk siblings”, “breastfeeding app or system” were considered. Initial investigation discovers that research on human milk exchange is mostly related to human milk banks. Boolean operators were used to combine the search strings. In this study, relevant studies were identified by adhering to the PRISMA Extension for Scoping Reviews (PRISMA-ScR) framework. The PRISMA-ScR flowchart diagram (refer Fig 1) was used as a guide to record the review process. The initial search results from databases yielded 360 articles. Thirteen articles which were not in English or Malay language as well as not related to the research objectives were directly excluded.

### Stage 2: Study selection and screening

To expedite the screening process, the RIS bibliographic citation file from scholarly databases were imported Rayyan[13]. Eighty seven duplicate articles were detected and filtered by the software. Then, two authors collaboratively reviewed the articles’ title and abstract based on the eligibility criteria (Table 1).

**Table 1:** Article eligibility criteria.

Inclusion Criteria	Exclusion Criteria
• Articles published in English or Malay • Articles published between 1^st^ January 2018 and 31^st^ December 2022 • Research focused on systems/applications related to breastfeeding, milk sharing, milk exchange, milk siblings/kinship • Articles published as original articles, reviews or conference proceedings	• Articles which are not technology related • Articles which are other than human milk • Articles which members have no access to the full text

The review process consisted of two levels of screening: (1) a title and abstract review and (2) a full-text review. For the first screening level, the titles and abstracts of articles retrieved in the search will be read and analysed by two independent investigators to identify potentially eligible articles. Nineteen articles were further excluded since the articles were not related to human milk. In the second step, two investigators independently assessed the full-text articles to determine whether the article met the inclusion and exclusion criteria. Any discordant full-text articles were reviewed a second time, and further disagreements about study eligibility at the full-text review stage were resolved through discussion with a third investigator until full consensus was obtained. Authors thoroughly examined the full texts of 183 articles to confirm that the articles fulfil the inclusion criteria. Finally, 78 articles fulfil the inclusion criteria of this study. The two-level screening procedure allows for incremental refining of the article selection, beginning with a broad scope and gradually limiting it down to those that meet the review’s particular eligibility requirements. It is also to avoid non-inclusion of potentially relevant articles in this study. The results of the selection according to the PRISMA flow diagram are shown in Fig 1.

### Stage 3: Charting and data analysis

**Fig 2:**
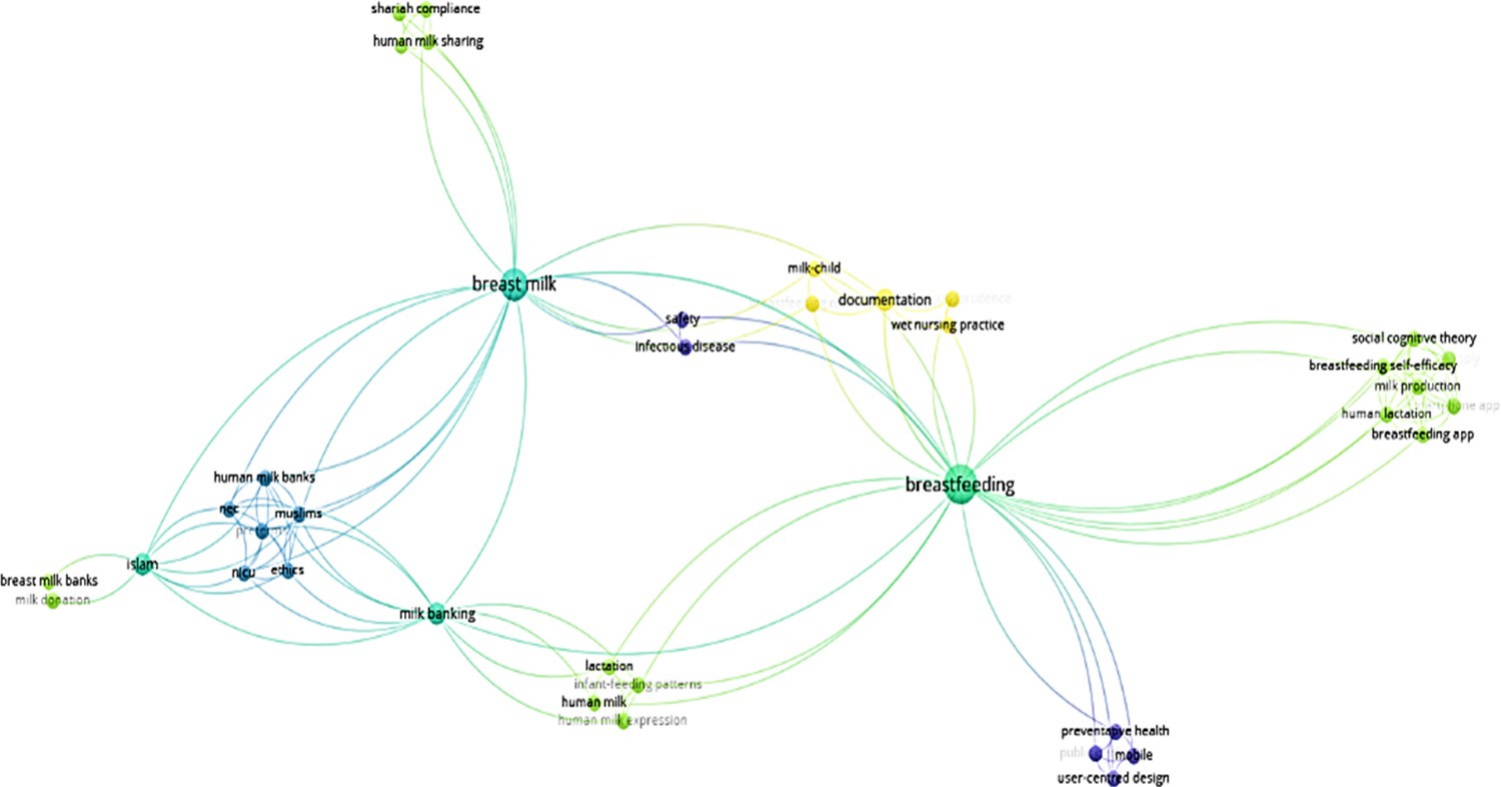
Co-occurrence analysis of the keywords (n = 78) (min 5 occurrence of all keywords).

The bibliometric data of selected articles were analysed with the aid of VOSviewer version 1.6.7 [14] based on co-citation analysis to designate groups of related texts using textual information. Collaborative network maps were generated to reveal the association of the articles in breastfeeding practice (Fig 2). The included articles were thoroughly read, and the data were summarized into a table.

## Results and discussions

The articles are clustered into five main groups related to breastmilk, breastfeeding and milk banking as in Fig 2. Articles which discussed human milk sharing focused on issues related to milk bank, Muslim community, and ethics when dealing with pre-natal care. Previous researchers also worked on breastfeeding practices in relation to social cognitive theory, lactation and milk production as well as breastfeeding app. Documentation issue was investigated in line with wet nursing practice and the impact on milk child relations.

Digital technologies through web-based interventions, mobile apps, and computer kiosks are used to acquire, manage, record and exchange information which generally promote breastfeeding practices. These technologies can further be categorized into six categories i.e., monitoring, and breastfeeding tracking, personalization, online discussion forum, web-based consultation, and breastfeeding station locator. Nevertheless, mobile applications use to support breastfeeding practices is appealing due to their relative ease of use and constant availability [12]. Moreover, study about the common features in breastfeeding mobile applications has been conducted in [15]. We summarized the breastfeeding applications from our findings in Table 2.

**Table 2:** Breastfeeding applications proposed by previous work.

Source	Country	Description	Modules
GMilk [7]	Philippines	To facilitate milk donation and requests, this study presents a framework for mobile and web applications where donors and requestors can easily search the nearest milk recipients, request and donate breastmilk with a validation process to address the gaps.	• Milk donation • Milk request • FAQs module • List of Milk Banks • Forum • Events • Articles • Milk Transactions • Profile • Applicant • Dashboard module (web only) • User Account Management (web only)
MilkTrack [16]	Philippines	A logistic system for the proper collection, transportation, and delivery of donated breastmilk to the recipients.	• Information and tutorials about breastfeeding • Breastfeeding station locator
KULEA-NET [17]	Columbia	A prototype mobile breastfeeding app guided by the preferences of African American parents.	• local resources • support person access • baby care logs, • identification of public breastfeeding venues • peer discussions
MommaMae [18]	Thailand	A mobile app aimed to support breastfeeding mothers to breastfeed in public and track their feeding and pumping logs effectively.	• Feeding record • Pumping record • Feeding rooms locator
Direct-to-consumer (DTC) Tele-lactation App [19]	USA	Aimed to increase access to International Board-Certified Lactation Consultants (IBCLCs) in rural settings.	• Two-way video through personal devices
BEST4Baby app [20]	India	A mobile app designed to supplement peer counselors’ training which is accessible during and after trainings as well as out in the field.	• logging in • Scheduling visits • Practicing visit • Content of the training modules
Milk Box tool [21]	USA	The tool was developed to capture real-time milk feeding type at every medical visit for children aged 0 to 2 years and incorporated into nursing workflows.	• Infant details • Milk feeding time • Milk feeding amount
CuidarTech Doe Leite [22]	Brazil	An app developed to manage human milk collection by administering the communication and interaction between Human Milk Bank (HMB) teams and donors as well as educate users on human milk donation.	• Profile • My donations • Home collection • Information to donate human milk • Contact the Human Milk Bank
RESTful Web Service [23]	Indonesia	The design of integrated information system prototype by integrating breast milk donor data obtained from human milk banks throughout the country.	• Donor registration • Recipient registration • API key generator • Breastfeed mother-child certificate
Milky Way [24]	Australia	Disseminating health information about breastfeeding benefits, challenges, and management strategies to the general community.	• Preparation • Milk Supply • FAQ • Ask ABA • Setting • Support
Breastfeeding Solutions [25]	Australia	The app was an interactive guide to resolve breastfeeding problems, provide functions for searching for problem solutions, and deliver timely information for mothers.	• Searching • Breastfeeding info

Moreover, we reviewed available applications using the search terms “breastfeeding”, “milk”, “lacta” and “*susu*” in the Apple App Store and Google Play Store. We discovered 41 breastfeeding apps and collaboratively examined the apps features. We found that among the features included in these applications are like baby sound recorder, baby’s photos gallery, bottle-feeding timer, breastfeeding timer, breast-pumping timer, diaper-change maker, baby’s sleep pattern, to-do list, baby’s mood, allergy, doctor visits, baby’s growth, milestones, baby’s temperature, baby’s medication tracker, baby’s teeth growth, weight history and multiple birth record. In addition, these applications are developed for the Western countries. None of the applications records informal human milk exchange or enable milk siblings’ traceability. Examples of these applications are listed in Table 3.

**Table 3:** Example of breastfeeding application in Google PlayStore.

Application	Descriptions
e-Anak Susuan Terengganu	• A mobile app to register breastfeeding child details and check status of the child. • Provide printable *mahram* certificate. • Provide quiz questions related to breastfeeding. • User can provide suggestions.
ICareMum’sMilk	Provide a platform for users and domain experts to share knowledge in the form of questions and answers on breastfeeding and lactation in Islamic doctrine. • Record donor and receiver details as well as breastfeeding child.
Milk Matters	• Donors may track their donations and an estimate of how many babies their milk will feed. • Provide donors with useful breastfeeding information, • Provide details about Milk Matters depots • Provide simple tool to self-assess their ability to meet certain essential requirements of donating breast milk. • Donors can contact Milk Matters through the app for further information.
Lactashare	• Developed by Indonesia developers • Provide registration platform for donor and receiver. • Provide online and offline lactation consultations • Receive donation to support related activities towards the development of Indonesia Milk Bank.
Milk Donor App	• Funded by the Nesta ShareLab grant • Facilitate the collection of donated breast milk by volunteer couriers to be processed at the Hearts Milk Bank (HMB)
Milk Messenger	• Verify donor status with phone number and donor ID verification • Add milk to freezer stash to track supply • Indicate when milk is sent, send notification to Mothers’ Milk Bank with information on incoming milk • Track milk en route • Track when milk is delivered • Order supplies from Milk Bank, submit order through email • Manage all account data, verify with Milk Bank through email
Mothers Milk	• Donor can volunteer to donate the milk and register as Donors. • The receiver can login and search the donor or the Milk Bank available nearest to his or her residence.
Breast Milk Donor App for Mothers’ Milk Bank Austin	• Provides prescribed donor human milk. • Provides information on breast milk and the effects on premature babies. • User can choose to donate, join a newsletter, or share info with other moms

We further investigate the Social Networking Sites, focusing on human milk exchange initiatives in Malaysia. In general, hospitals have launched human milk exchange programs to aid premature infants and mothers. Records are exclusively kept and maintained by the hospitals. Recently, the first syariah-compliant bank was launched in Malaysia, known as Bonda Halimatussaadia Milk Center [26]. Table 4 lists examples of approaches by Non-Governmental Organization (NGO) support groups to facilitate informal human milk exchange through social networking sites.

**Table 4:** Examples of NGO support groups.

Support Group	Approach
Johor Bahru Breastfeeding Moms Support Group (*Kelab Sokongan Penyusuan Susu Ibu Johor Bahru*)	FB page visitors can view a spreadsheet of registered applications from both donor and biological parents/guardian of infants (seeker). If there are no potential candidates, the donors/seekers are required to fill-up their contact details and answer a few questions related to their intention in a Google Form. On the contrary, once a suitable donor/seeker is identified, communication between both interested parties may be initiated. The page owner is not responsible to keep records and manage the relationship between both parties
Kedah’s Breastfeeding Mothers Support Group (*Persatuan Kumpulan Sokongan Penyusuan Susu Ibu Negeri Kedah–PKSPK*)	Enable search announcement/ news posts as follows: • Searching for non-maternal nursing mother • Offering excessive expressed breastmilk (EBM) Further actions are not facilitated by the group.
Human Milk 4 Human Babies (HM4HB) (http://www.hm4hb.net/index.html)	Aims to facilitate human milk exchange among members from multiple race and religion. Use Google Form to share details of donor and seeker. Registration details are accessible publicly in the form of Google Sheet, including name, location/state, profile page, contact number and e-mail address. Records from the Google Sheet will be removed once prompted by the data owner (either donor or seeker).

The challenge encountered by this scoping review was the limited number of publications available on technology-based approaches for informal human milk sharing. In order to be as thorough as possible, we gathered data from the Google Play Store as well as Facebook social media groups which are publicly accessible. Moreover, since this scoping review does not typically evaluate the quality of individual studies, the findings may not be applicable to all contexts. However, the review is appropriate to achieve our research objectives.

## Conclusion

This study makes an important contribution to the literature by identifying the main clusters of scholarly articles related to breastfeeding from year 2018–2022. We found that most scholarly works focused on breastmilk, breastfeeding and milk banking challenges and issues with recurrent themes i.e., societies, lactation inadequacy and unresolved nursing problems. In addition, this study reviewed technologies and application presented during the same period to encourage breastfeeding practices. Available mobile applications were also reviewed based on the features offered.

Moreover, in certain nations, milk kinship remains a major concern especially among WEIRD societies. This has hindered the acceptance of human milk bank. However, since it is a well-known fact that human breastmilk is the best source of nutrition for infants, informal human milk exchange is a feasible option for mothers who cannot breastfeed their child or have insufficient supply, unfortunate infants with deceased mother or premature infants. Even so, informal human milk exchange which takes place locally among family members, relatives and friends are less known [6]. In Malaysia for example, social networking sites have also been used to facilitate informal milk exchange between the donor and recipient through news posts and requests made using Google Forms. Proper documentation and recording of the actual informal human milk exchange relies on the awareness of the donor and recipient (biological parents/guardian) to record the engagement. Hence, effort to increase the awareness of the society on the impact of milk kinship must be continuously made as well.

## Supporting information

S1 Checklist Preferred Reporting Items for Systematic reviews and Meta-Analyses extension for Scoping Reviews (PRISMA-ScR) checklist. (DOCX)
